# Undertriage in trauma: an ignored quality indicator?

**DOI:** 10.1186/s13049-020-00729-6

**Published:** 2020-05-06

**Authors:** Elisabeth Jeppesen, Mathias Cuevas-Østrem, Cathrine Gram-Knutsen, Oddvar Uleberg

**Affiliations:** 1 Department of Research and Development, Norwegian Air Ambulance Foundation, NO-0103 Oslo, Norway; 2 Faculty of Health Science, University of Stavanger, Stavanger, Norway; 3 Department of Emergency Medicine and Pre-Hospital Services, St. Olav’s University Hospital, NO-7006 Trondheim, Norway; 4 Department of Circulation and Medical Imaging, Norwegian University of Science and Technology, NO-7006 Trondheim, Norway

**Keywords:** Trauma system, Undertriage, Quality indicator

## Abstract

**Background:**

Early identification of life-threatening injuries is essential to reduce morbidity and mortality in trauma patients. Failure to detect severe injury may cause delayed diagnosis and therapeutic interventions and is associated with increased morbidity. A national trauma system will contribute to ensure the optimal care for seriously injured patients throughout the treatment chain by, among other things, defining a sensitive triage tool for identifying severe injury and contribute to correct treatment destination. In 2017, a National trauma plan was implemented in Norway and several quality indicators were recommended to ensure an evaluation of potential gaps between achieved and desired quality, and thereby highlighting areas with potential for quality improvement. With this commentary, we want to draw attention to, what we believe is, an ignoring of an important quality indicator: undertriage in trauma.

**Main body:**

Severely injured patients not met by a trauma team is commonly referred to as undertriage. An undertriage rate below 5 % is an internationally recognized quality indicator in trauma care and is emphasized in the Norwegian national trauma plan. However, whether hospitals measure and report data about undertriage, have received little attention. Therefore, a national survey was performed among Norwegian hospitals, where thirty-seven of forty trauma receiving hospitals contributed. The results of the survey showed that only half of Norwegian trauma hospitals were capable of providing these data. The results of this survey show that currently the national trauma system is not equipped to obtain important data on an important and specific quality indicator. An ongoing discussion at a national level is how to define severe injury, which may alter future definitions on undertriage.

**Conclusions:**

Knowledge of undertriage in trauma is important to enhance patient safety, increase the precision of the triage tool and provide valuable learning information to individual hospitals and prehospital services. Currently only half of Norwegian hospitals who receive trauma patients report undertriage rates and unfortunately, only few hospital administrators request these data.

## Background

Early identification of life-threatening injuries is essential to reduce morbidity and mortality [1]. Triage is used at the scene for multiple purposes; to separate patients with potentially serious injuries from those with minor injuries, prioritizing patients for adequate transport, choosing the right destination and finally choosing the correct response at the receiving hospital. Sensitive triage tools which include physiologic variables, specific anatomic injuries, mechanism of injuries and certain special causes (e.g. age and severe co-morbidity) assist prehospital personnel in the triage process [1]. Suspicion that a person is seriously injured is based on all available knowledge - but due to the need of rapid transportation and treatment, the decision to consider the patient as seriously injured is often based on limited information. Patients who are severely injured (Injury Severity Score [ISS] greater than 15) and not received by a trauma team are commonly referred to as undertriaged (false-negatives) [2, 3]. Failure to detect severe injury may cause delayed diagnosis and therapeutic interventions and is associated with increased morbidity [4, 5]. Conversely, considering a patient to be severely injured where there appears to be less severe injury (ISS < 15), is defined as overtriage. The overtriage rate is defined to capture the proportion of unnecessary use of hospital resources on minor trauma (resource overutilization) and could create concurrent conflicts for treating health care professionals with adverse consequences for other patients [6].

An undertriage rate of < 5% is often considered as acceptable according to the American College of Surgeons Committee on Trauma (ACS-COT) [1]. However, previous Norwegian studies have shown substantial higher rates of undertriage at Norwegian hospitals, varying between 13 and 28% [7–9], with similar rates seen in systems in the Netherlands and the United States [10, 11]. Therefore, undertriage in trauma is still a challenge even for the most highly developed trauma systems [12].

A trauma system should help to ensure that seriously injured patients are optimally cared for throughout the treatment chain [1]. In 2017, a National trauma plan was implemented in Norway to ensure an optimal systematic approach and common practices to improve trauma management throughout the country [13]. Several quality indicators were recommended to ensure an evaluation of potential gaps between achieved and desired quality, and thereby highlighting areas with potential for quality improvement. One of the most important quality indicators was the rate of undertriage at Norwegian hospitals. An undertriage rate < 5% was seen as an important indicator of triage precision. However, whether hospitals themselves actually are capable and have the capacity to report these and other quality indicators, have received all too little attention. With this commentary, we want to draw attention to, what we believe is, an ignoring of an important quality indicator: undertriage in trauma, defined as patients with ISS > 15 and not received by a trauma team.

## Main text

A national survey was performed where a questionnaire was sent to certified registrars in all hospitals reporting data to the Norwegian Trauma registry (NTR) in 2017. NTR should include all patients with New Injury Severity Score [NISS] > 12, head injury AIS ≥ 3 and patients with penetrating injuries proximal to elbow or knee, regardless of whether they have received by a trauma team or not. The registrars were asked if they identify and include patients without trauma team activation in the registry. Thirty-seven of forty hospitals, including all four trauma centres, responded to the survey. Nineteen hospitals (51%) stated that they identified and reported undertriage rates. According to results from open questionnaires in the survey, lack of time and resources (57%) were identified as the main barrier to not be able to identify undertriage. Secondly, the working process of retrieving these data was labour-intensive as no precise and adequate technical solutions were available for the data extraction. In addition, half of the hospitals (19 of 37) stated that data on undertriage was not requested, or of interest, by their own hospital administration. The results of the survey shown that only half of Norwegian trauma hospitals were capable of providing data on a nationally defined quality indicator on trauma management.

A study by Wisborg et al., found that only 50% of seriously injured patients in Norway are offered advanced anaesthesiology-led care outside hospitals. The combined effect of both pre- and in-hospital undertriage may lead to negative patient outcome [14]. In a study by Voskens et al., the undertriage rate among older adults were 38.6% [10]. This result highlights the specific challenges of increased undertriage seen in the older population. Experiences from clinical practice show that undertriaged geriatric patients with low-energy trauma can have few presenting complaints and clinical findings despite severe injuries, typically in the head or as occult internal bleedings. In the absence of red flags, the acuity is low, and they are often evaluated in a community casualty clinic before presenting to hospital. As the current triage algorithms were primarily developed based on a younger trauma population, the increased cohort of older trauma patients with subtle injuries underlines the increased need attention on undertriage rates [12].

Throughout many years the ability of field triage criteria to identify precisely severely injured patients have been addressed [2]. The results of this survey show that currently the national trauma system is not equipped to obtain important data on an important and specific quality indicator. An ongoing discussion at a national level is how to define severe injury, which may alter future definitions on undertriage. Today, the most commonly used indicator of severity is the ISS, but variables such as the New Injury Severity Score (NISS), length of hospital stay and performed hospital interventions have also been proposed as measures of severity [15]. Though irrespective on how we define severity, the ability to measure and provide a follow-up on an established quality indicator is imperative for improvement initiatives. A lack of follow-up may lead to the disregard of undertriage as a quality indicator.

## Conclusions

Knowledge of undertriage in trauma is important to enhance patient safety, increase the precision of the triage tool and provide valuable learning information to individual hospitals and prehospital services. Currently, only half of Norwegian hospitals who receive trauma patients report undertriage rates and unfortunately, only a few hospital administrators request these data. Lack of time, resources and adequate technical solutions are the main challenges in order not to prioritize this task.

## Data Availability

The datasets used during the current study are available from the corresponding author on reasonable request*.*

## Abbreviations

ISS: Injury Severity Score
NISS: New Injury Severity Score
NTR: Norwegian Trauma Registry
